# Gene expression variation between mouse inbred strains

**DOI:** 10.1186/1471-2164-5-57

**Published:** 2004-08-18

**Authors:** Rolf Turk, Peter AC 't Hoen, Ellen Sterrenburg, Renée X de Menezes, Emile J de Meijer, Judith M Boer, Gert-Jan B van Ommen, Johan T den Dunnen

**Affiliations:** 1 Center for Human and Clinical Genetics, Leiden University Medical Center, Wassenaarseweg 72, 2333AL Leiden, Nederland; 2 Department of Medical Statistics, Leiden University Medical Center, Wassenaarseweg 72, 2333AL Leiden, Nederland

## Abstract

**Background:**

In this study, we investigated the effect of genetic background on expression profiles. We analysed the transcriptome of mouse hindlimb muscle of five frequently used mouse inbred strains using spotted oligonucleotide microarrays.

**Results:**

Through ANOVA analysis with a false discovery rate of 10%, we show that 1.4% of the analysed genes is significantly differentially expressed between these mouse strains. Differential expression of several of these genes has been confirmed by quantitative RT-PCR. The number of genes affected by genetic background is approximately ten-fold lower than the number of differentially expressed genes caused by a dystrophic genetic defect.

**Conclusions:**

We conclude that evaluation of the effect of background on gene expression profiles in the tissue under study is an effective and sensible approach when comparing expression patterns in animal models with heterogeneous genetic backgrounds. Genes affected by the genetic background can be excluded in subsequent analyses of the disease-related changes in expression profiles. This is often a more effective strategy than backcrossing and inbreeding to obtain isogenic backgrounds.

## Background

Due to their isogenicity, inbred mouse strains demonstrate low biological variability within each strain[1,2]. Genetic variation between inbred strains is considerable and has recently been characterized in detail using single nucleotide polymorphisms[3]. Differences in genetic background between strains affect the gene expression levels of a subset of genes, which probably explains phenotypic differences. Indeed, several reports have been published in which gene expression profiles have been used as QTLs in genetic mapping studies to identify complex traits [4-6].

From literature [7-9], it appears that the subset of genes for which expression is significantly affected by genetic background is small. However, this has never been related to the extent of gene expression changes observed due to disease-causing mutations. We are studying differential gene expression between affected and healthy muscle in a range of murine models for neuromuscular disorders with different genetic backgrounds (Turk *et al*., manuscript in preparation). We, therefore, determined gene expression levels in hindlimb muscles from five frequently used wildtype mouse inbred strains, and compared these to the differential gene expression levels in affected muscle tissue from a mouse model (*mdx*) for Duchenne muscular dystrophy with healthy muscle tissue. Both the number of differentially expressed genes between strains as well as the fold-change levels are lower when compared to the differences found in affected versus healthy muscle tissue.

## Results

Gene expression levels in hindlimb muscle tissue from five different inbred strains (CBA, BALB, BL6, DBA, and BL10) were determined. Total RNA from two individuals per strain was isolated, reversed transcribed, and subsequently labelled according to a recently developed protocol (adapted from Xiang *et al.*, 2002), which requires an input of only 1 μg total RNA. Labelled cDNA was hybridised to murine microarrays containing 7,776 65-mer oligonucleotides spotted in duplicate.

Significance levels (p-values) between the five mouse inbred strains were calculated using analysis of variance[10]. Significance levels among two individual mice within each strain were determined using a hierarchical *t*-test providing higher statistical power than conservative methods for low (2–4) replicate numbers[11]. The higher power is yielded by borrowing information across genes to produce a better expression variance estimator. The gain in power is reported via an increase in the degrees of freedom associated with the t-test. Differentially expressed genes for both computations were selected by controlling the false discovery rate (FDR), as suggested by Benjamini and Hochberg (1995), rather than using pre-defined cut-offs for p-values or corrections for multiple testing. The FDR represents an expectation of the proportion of false positives among the selected differentially expressed genes, which increases dramatically during multiple testing, inherent in microarray experiments[12].

**Figure 1:**
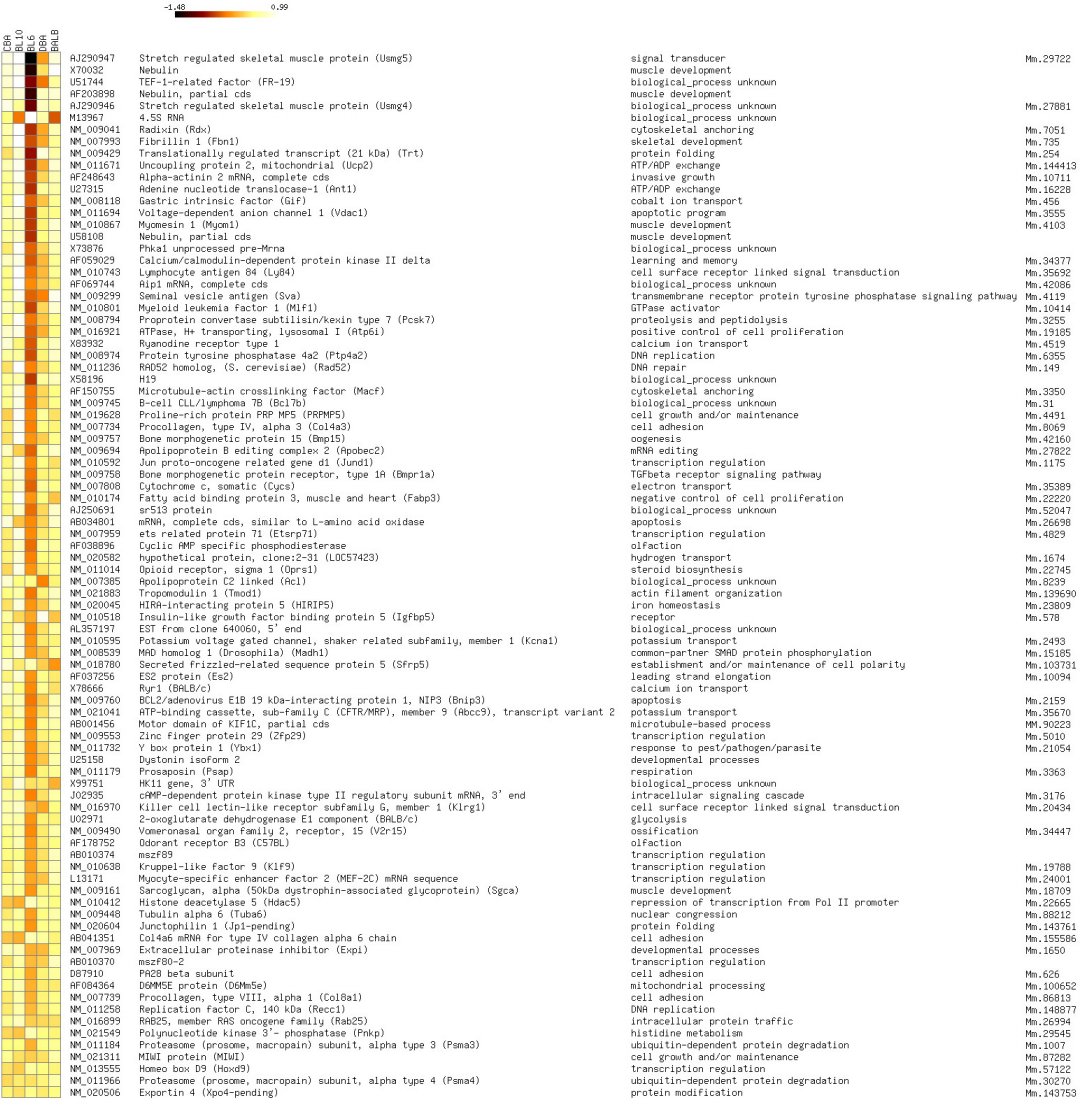
Differentially expressed genes between mouse inbred strains Relative expression levels of differentially expressed genes between mouse inbred strains are depicted in colour as relative intensity levels. Shown for each gene are GenBank accession number, description, functional annotation according to Gene Ontology, and UniGene cluster IDs. Relative expression levels are calculated by subtracting the average intensity value per gene from the strain-dependent intensity values. Differential expression was determined by selecting p-values from analysis of variance based on a false discovery rate of 10%.

Using an FDR of 10% we selected 88 out of 6144 (1.4%) expressed genes that are differentially expressed between strains (Fig. 1). A lower number of differentially expressed genes was found in the analysis of variation within strains with identical FDRs of 10% (Table 1). Results with other FDR levels are available online as additional file. Correlation between gene expression levels of the two samples from each strain was high (Pearson correlation coefficient ranging from 0.87 to 0.95), also indicating low internal variation (Table 1). A considerable amount of differentially expressed genes (718 genes) were selected when pre-defined cut-off values (p < 0.05) were used to determine the differential gene expression between strains. However, adjusted FDR levels indicated a proportion of false positives equal to 42%. On the other hand, adjusting for multiple testing using Bonferroni correction proved to be too stringent, leaving no or few differentially expressed genes. Controlling the FDR, therefore, appears to be an optimal method for both selecting differential gene expression and simultaneously determining the validity of the experimental outcome.

**Table 1:** Number of differentially expressed genes using several cut-off strategies

	**Between strains**	**Within strains**				
	MA-ANOVA	Hierarchical *t*-test				
	
		**CBA**	**BL10**	**BL6**	**DBA**	**BALB**
Correlation		0.95	0.95	0.87	0.87	0.92

Naive (p < 0.05)	718	737	610	963	1043	483
Bonferroni	0	2	4	1	0	3
FDR 10%	88	2	4	14	0	16

Correlation between two individuals per strain was calculated using Pearson's correlation coefficient. Significance levels (p-values) between strains were calculated with MA-ANOVA, and within strains using the hierarchical *t*-test. Differential gene expression was determined by selecting genes with p-values lower than a specified threshold. Thresholds were selected using three different strategies; naive, Bonferroni corrected, and False Discovery Rate (10%), and resulted in different numbers of significantly differentially expressed genes.

**Figure 2:**
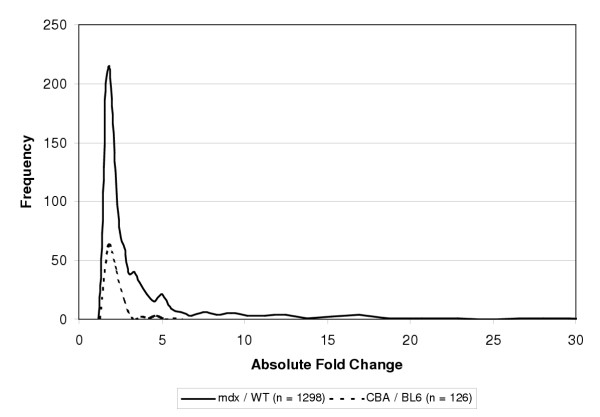
Effect of different genetic background on differential gene expression The distribution of absolute fold changes of differentially expressed genes (n = 1298) between affected (*mdx*) and healthy (WT) muscle were compared to the distribution of absolute fold changes of differentially expressed genes (n = 126) between two mouse inbred strains (CBA and BL6). Selections were based on a FDR of 10%.

To put the influence of differential gene expression due to genetic background in perspective, we studied gene expression between affected and healthy tissue from hindlimb muscle derived from *mdx *mice, and from control mice with identical genetic backgrounds. Selection with an FDR of 10% resulted in 1298 differentially expressed genes. Differential gene expression between the two most divergent mouse inbred strains (BL6 and CBA, data not shown) was determined to allow a direct comparison with identical statistical methods. Selection with an FDR of 10% showed an approximately ten-fold decrease in the number of differentially expressed genes (126). Absolute fold changes were calculated and subsequently a comparison of the distribution was made (Fig. 2). Median gene expression levels are equal between affected/control and inbred/inbred. However, the number of large fold changes (>3) between affected/healthy (221) is much higher than between inbred/inbred (7), consistent with low contribution of differential expression due to genetic background.

**Figure 3:**
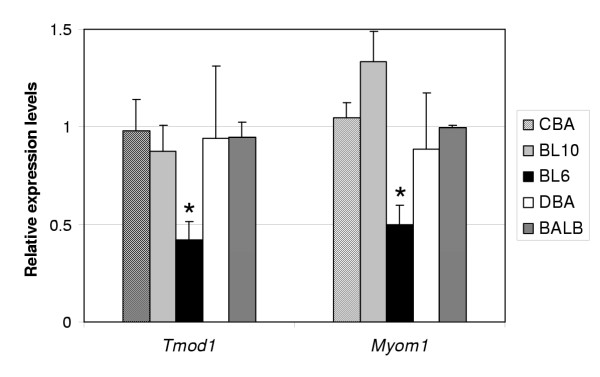
Validation of BL6-dependent gene expression with qPCR Relative gene expression levels between mouse inbred strains of tropomodulin 1 (*Tmod1*) and myomesin 1 (*Myom1*) as determined by quantitative RT-PCR. Significantly lower expression (p < 0.01, marked by *) for both genes was shown in BL6 compared to other strains.

Although overall expression levels are similar between strains, a relatively high number of differentially expressed genes was due to deviating gene expression levels in BL6. We performed quantitative real-time RT-PCR (qPCR) on five genes to verify our microarray data. Two genes myomesin 1 and tropomodulin 1, which were 2.2-fold and 1.8-fold lower expressed in BL6 compared to the other strains on our microarrays, were also found to be lower expressed (2.0-fold and 2.2-fold respectively) in our qPCR assay (Fig. 3). Three other genes (dysferlin, cystatin B, and thrombospondin 4) showed no differential expression between any strains.

## Discussion

This study shows that variation in overall gene expression levels between mouse inbred strains is relatively low in hindlimb muscle tissue. This is particularly evident when the number of differentially expressed genes between two mouse inbred strains (C57 vs. Bl6, 126 genes with 7 genes having a fold-change > 3) is compared to that between diseased and healthy muscle tissue (*mdx *vs. wild-type, 1298 genes with 221 genes having a fold-change >3). Therefore, the use of mice with deviating genetic background may be justified in disease-related studies. Alternatively, strain-dependent gene expression differences may be evaluated in the initial study phase of gene targeting experiments, although the effect of hybrid backgrounds is difficult to assess.

Gene expression studies in the brain revealed that approximately 1% of expressed genes differ between two mouse strains[8]. Application of alternative statistical methods, similar to those used in our study, on this dataset resulted in an increase in the number of differentially expressed genes (approx. 3%) between the two mouse strains[7], demonstrating that the number of differentially expressed genes is highly dependent on the statistical criteria used. A similar number of differentially expressed genes was found in a comparison of hippocampal gene expression between 8 different mouse strains[9]. The results of our study in muscle tissue demonstrated that approximately 1.4% of the expressed genes show differential expression between mouse strains. Based on these results, strain differences in gene expression seem to have a similar magnitude across different tissues.

Genomic variability could be correlated with high levels of single nucleotide polymorphisms (SNPs) occurring in specific blocks between mouse inbred strains. The presence of cis-acting (single nucleotide) polymorphisms may be associated with regulatory variation affecting gene expression levels. It was estimated that probably a consistent amount (up to 6%) of the roughly estimated 35,000 mouse genes contain such functional regulatory variants[13]. We investigated if differentially expressed genes were localized in blocks with high genomic variability, but our number of differentially expressed genes was too low to obtain statistically significant answers (data not shown).

This study suggests an additional method for phenotyping mouse inbred strains and provides a list of genes with significant differential expression based upon false discovery rate selection. Although overall gene expression profiles are highly similar, most significant differences are determined by low gene expression levels of BL6 compared to the other strains. A large proportion of these BL6-specific genes function as structural muscle proteins (i.e. nebulin, alpha-actinin 2, myomesin 1 and radixin). To date, however, no major differences in muscle physiology in BL6-mice have been described which can be attributed to these reduced gene expression levels.

Perfectly isogenic backgrounds are sometimes difficult to obtain. This explorative study demonstrates that the effect of genetic background on muscle expression profiles is significant but rather limited compared to other effects, e.g. the dystrophic genetic defect (*mdx*) we study. As such, the genetic background will only marginally interfere with data analysis. Determination of gene expression profiles between mouse strains enables flagging a modest number of differentially expressed genes, and is an efficient and sensible approach to circumvent tedious backcrossings, necessary to obtain isogenic animals.

## Methods

### Mouse breeding, tissue preparation and total RNA isolation

We obtained CBA/CaOlaHsd (CBA), BALB/cOlaHsd (BALB), C57Bl/6JOlaHsd (BL6), DBA/2OlaHsd (DBA), and C57Bl/10ScSnOlaHsd (BL10) mice from Harland Laboratories, and C57Bl/10ScSn-Dmd^mdx/J ^(*mdx*) mice from Jackson Laboratory at the age of 6 weeks. Mice were kept under standard conditions and were sacrificed by cervical dislocation when 8 weeks old. Hindlimb muscles (m. quadriceps femoris) were dissected and promptly snap-frozen in isopentane at -80°C. Total RNA was prepared by disrupting tissue using mortar and pestle and subsequent homogenisation by a rotor-stator homogenizor (Ultra-Turrax T25, Janke & Kunkel IKA-Labortechnik) in RNA-Bee (Campro Scientific) until uniformly homogenous (15–45 sec). Total RNA was isolated according to manufacturer's instructions followed by purification using RN-easy columns (Qiagen). Quality and yield was determined using Lab-on-a-chip (BioAnalyzer, Agilent).

### Target preparation and hybridisation

Aminoallyl labelled cDNA (aa-cDNA) was prepared based on a previously described protocol[14]. Aliquots of 1 μg of total RNA in the presence of 2 μg amino-TN_6 _primer (5'-NH_2_-(CH_2_)_6_-TN_6_, Eurogentec) were adjusted to a volume of 21 μl with DEPC-treated H_2_O (diethyl pyrocarbonate, Sigma), heated for 10 minutes at 70°C and chilled on ice for 10 minutes. Reverse transcription mastermix (1.8 μl RevertAid RNaseH-M-MuLV reverse transcriptase (200 U/μl, MBI Fermentas), 6 μl 5x first-strand buffer (MBI Fermentas), and 1.2 μl 25x aa-dUTP / dNTP solution (2 μl 50 mM dATP, 2 μl 50 mM dCTP, 2 μl 50 mM dGTP, 1.2 μl 50 mM dTTP, 0.8 μl 50 mM aminoallyl-dUTP (Ambion)) was added per reaction and incubated at room temperature for 10 minutes followed by 2 hours at 42°C. RNA was hydrolysed by addition of 10 μl 0.5 M EDTA and 10 μl 1 M NaOH and incubation at 65°C for 30 minutes followed by neutralization by addition of 10 μl 1 M HCl. Aminoallyl labelled cDNA was then purified by combining 300 μl of PB-buffer (Qiagen) to 60 μl of the neutralized sample and centrifuged through a Qiaquick column (Qiagen) at 13000 rpm for 1 minute. Two washing steps were performed by spinning 500 μl of 75% EtOH at 13000 rpm for 1 minute while discarding the flow-through. To remove ethanol-traces the columns were centrifuged for an additional minute. cDNA was recovered by eluting three times using 30 μl basic H_2_O (3.3 mM NaHCO_3 _buffer, pH 9.0) and concentrated to a volume of 6.66 μl using a speedvac. Aliquots of Cy3 and Cy5 reactive dyes (PA23001, PA25001, Amersham) were prepared by dissolving each vial of monoreactive dye in 40 μl fresh anhydrous DMSO (Sigma) and dividing into aliquots of 2 μl followed by vaccuumdrying until dry and subsequent storage at 4°C in the presence of silica. Fluorescent dyes were coupled by adding 3.33 μl of bicarbonate buffer (1 M NaHCO_3 _buffer, pH 9.0) to the aa-cDNA sample and dissolving the dried aliquot of reactive dye, followed by incubation at room temperature for 1 hour in the dark. To the samples 4.5 μl 4 M hydroxylamine (Sigma) was added and incubated at room temperature in the dark for 15 minutes, followed by addition of 186 μl TE^-3^-buffer. Hybridisation mixtures were prepared by combining a Cy3-labeled cDNA sample with a Cy5-labeled cDNA sample and 10 μl Mouse-Hybloc (1 μg/μl, Applied Genetics Laboratories) followed by removing uncoupled dyes by spinning through a pre-wetted Microcon column (YM30, Amicon) for 8 minutes at 13000 rpm. Hybridisation mixture was washed by spinning 500 μl TE^-3^-buffer through the column and discarding the flow-through. This step was repeated two times as 2 μl yeast-tRNA (10 μg/μl, Sigma) and 2 μl polyA-RNA (10 μg/μl, Sigma) were added during the last step. Mixture was collected by inverting the column and spinning for 1 minute at 13000 rpm. Hybridisation mixture was finalized by adding TE^-3^-buffer to 84 μl together with 17 μl 20x SSC and 3 μl 10% SDS followed by denaturing at 100°C for 2 minutes, renaturing at room temperature for 15 minutes and spinning at 13000 rpm for 10 minutes. Labelled target was hybridised overnight on murine oligonucleotide microarrays (65-mer with 5'-hexylaminolinker, Sigma-Genosys mouse 7.5 K oligonucleotide library, spotted in duplicate). Hybridisation occurred in a automatic hybridisation station (GeneTac, Perkin Elmer) and was followed by washing with 5x 2xSSC + 0.1% SDS at 30°C, 5x 1xSSC at 30°C, 3x 0.2xSSC at 30°C, 1x 0.2xSSC at 65°C, 2x 0.2xSSC at 30°C, and subsequently scanned as described previously[15].

### Experimental design, data extraction and analysis

Gene expression profiles from hindlimb muscle derived from 2 male animals of each strain were generated using dye-swap experiments. Subsequent duplicate spots on each array resulted in 8 replicate measurements per gene. Targets were assigned at random to the arrays, while avoiding co-hybridisation of samples from the same strain. GenePix Pro 3.0 (Axon) was used for feature extraction and quantification. Genes were considered as being expressed when the corresponding feature was not flagged by the algorithm provided by GenePix. Local background corrected spot intensities were normalized using Variance Stabilization and Normalization (VSN) in R [16]. Array data has been made available through the GEO data repository of the National Center for Biotechnology Information under series GSE662. Correlation between individuals was calculated using Pearson's correlation coefficient. Significantly differential expression levels were determined using MA-ANOVA (**MAANOVA2.0 The Jackson Laboratory **), hierarchical *t*-test [11] and the False Discovery Rate [17] selection procedure.

### Quantitative Reverse Transcription Polymerase Chain Reaction

qPCR was performed in duplicate for each individual resulting in four measurements per strain per gene. cDNA was prepared by reverse transcription using 1 μg total RNA as template. Random hexamers (40 ng) were used to prime the transcription after heating 10 minutes at 70°C followed by chilling on ice for 10 minutes. cDNA was synthesized by RevertAid RNaseH^- ^MuLV reverse transcriptase and accompanying buffer (MBI-Fermentas) using 1 mM dNTPs. The mixture was incubated at room temperature for 10 minutes before a 2 hour incubation step at 42°C, followed by 10 minutes at 70°C. Quantitative PCR was performed using the Lightcycler (Roche). PCR mixture was prepared by combining cDNA dilution, 10 pmol forward and reverse primer, MgCl_2 _(4 mM) with 4x home-made LC mastermix (0.9 mM dNTPs, BSA (1 μl/μl, Pharmacia Biotech), Taq polymerase (0.8 U/μl), 4x SYBR Green I (Molecular Probes), 4x AmpliTaq Reaction Buffer (Perkin Elmer)) to a total volume of 20 μl. Amplicons were generated during 45 cycles with annealing temperature set at 55°C. Optimal cDNA dilutions and relative concentrations were determined using a dilution series per gene. Replicate experiments (n = 4) were normalized to 1 and relative expression values were determined by calculating the ratio per gene over the average relative expression of genes, which show no differential expression on both microarray and qPCR (dysferlin, cystatin B, and thrombospondin 4). Significance levels were calculated with a one-sample *t*-test. PCR primer pairs were designed using the Primer3 search engine, available at: **Primer3 Software Distribution **. The screened genes and the oligonucleotide primer pairs used for each of the genes in this study correspond to the following nucleotides: myomesin1, 4761–4780 and 4865–4884 (NM_010867); tropomodulin1, 670–689 and 878–897 (NM_021883); dysferlin, 4218–4237 and 4353–4372 (AF188290); cystatinB, 3–22 and 151–170 (NM_007793); thrombospondin4, 2167–2186 and 2289–2308 (NM_011582).

## Authors' contributions

RT carried out the tissue preparation, total RNA isolation, target preparation, hybridisations, experimental design, data extraction, data analysis, rt-PCR, and the drafting of the manuscript. PH participated in the experimental design, analysis, rt-PCR, and study coordination. ES participated in the experimental design and analysis. RM provided statistical support. EM was responsible for mouse breeding and tissue preparation. JM participated in experimental design. GO and JD coordinated the study. All authors read the final manuscript.

## Supplementary Material

Additional File 1 Differentially expressed genes between mouse inbred strains are selected with a false discovery rate of 10, 15, and 20%. Selected genes are indicated with 1, genes not selected by the specified criteria are indicated with 0. The mean of the relative gene expression levels of each of the five mouse strains is shown. For each gene the GenBank accession number is shown as well as the UniGene ID, gene description and the gene ontology description. The additional file is formatted as Comma Separated Values (CSV) file, and is named Turketal2004_Additional_File.csv. Click here for file
